# Analysis of additivity and synergism in the anti-plasmodial effect of purified compounds from plant extracts

**DOI:** 10.1186/1475-2875-10-S1-S5

**Published:** 2011-03-15

**Authors:** Eric Deharo, Hagai Ginsburg

**Affiliations:** 1 Laboratoire de Pharmacochimie des Substances Naturelles et Pharmacophores Redox, UMR 152, UPS, Université de Toulouse, 118 Route de Narbonne, F-31062 Toulouse cedex 9, France; 2 Institut de Recherche pour le Développement, UMR 152, Mission IRD casilla 18-1209 Lima, Peru; 3 Department of Biological Chemistry, Institut of Life Sciences, The Hebrew University of Jerusalem, Jerusalem 91904, Israel

## Abstract

In the search for antimalarials from ethnobotanical origin, plant extracts are chemically fractionated and biological tests guide the isolation of pure active compounds. To establish the responsibility of isolated active compound(s) to the whole antiplasmodial activity of a crude extract, the literature in this field was scanned and results were analysed quantitatively to find the contribution of the pure compound to the activity of the whole extract. It was found that, generally, the activity of isolated molecules could not account on their own for the activity of the crude extract. It is suggested that future research should take into account the “drugs beside the drug”, looking for those products (otherwise discarded along the fractionation process) able to boost the activity of isolated active compounds.

## Introduction

In the search for anti-malarial activity of plants traditionally used against fevers, collected plants are first submitted to an extraction process with polar or apolar solvents. Ideally, the extracts are then tested against erythrocytic stages of *Plasmodium falciparum in vitro* to validate anti-plasmodial activity. Classically, when biological tests identify significant activity, crude extracts are submitted to a bioguided fractionation procedure, aiming to isolate the active compound(s). For that purpose, several sequential extractions with solvents of diverse polarities are performed, and purified fractions are submitted to anti-plasmodial tests and to chemical identification. Frequently, many promising extracts are discarded because the anti-plasmodial activity disappears along the fractionation process. The failure to isolate active constituents from active extracts may be due to the lability/instability of the active compounds that are degraded during the extraction process. Sometimes, the loss of activity is due to the fact that the compounds display their activity only when they interact in the crude extract. Such compounds will be lost for further development unless their interactions can be examined. In order to evaluate such interactions, mostly synergistic, it is necessary to know the inhibitory activity of the crude extract and the purified fractions (i.e., their IC_50_ values) and the yields of extraction of the purified compounds to allow calculation of their absolute quantitative prevalence in the extract. Unfortunately, in most cases, when plants are extracted and fractionated, the activity of the crude extract is not determined and the yields are not reported or not determined altogether. This is the case of hundreds of thousands of purified fractions of natural extracts that have been evaluated by cell-based inhibition tests.

To determine the quantitative contribution of the pure compounds to the activity of a crude extract, data from the literature were compiled selecting those publications in which the activities of the crude extracts and of the purified compounds (and their yields) were reported. To calculate the contribution of the pure active compound to the activity of the extract, the respective IC_50_ values and the yield of the purified compound are used. Data are shown in table 1.

**Table 1:** Compilation of data from the literature on the anti-plasmodial effects of plant extracts and their fractionated active compounds. CS and CR are chloroquine-sensitive and –resistant strains respectively.

Plant Species	Family	Parasite strain	Extract IC_50_ μg/ml	Most active compounds*	Compounds IC_50_ μg/ml	Yield %	% of active comp of extract IC_50_	Contribution of active compound to extract inhib %	ref #
*Alstonia macrophylla* Wall.	Apocynaceae	*Pf* K1 CR	5,7	Macrocarpamine	0,27	0,95	0,05	33,4	1
*Alstonia macrophylla* Wall.	Apocynaceae	*Pf* K1 CR	5,7	Villalstonine	0,17	0,6	0,03	31,1	2
*Artemisia indica* Willd	Asteraceae	*Pf* K1 CR	6,6	Exigua flavanones	4,6	0,15	0,01	0,4	3
*Brucea javanica* L. (Merr.)	Simaroubaceae	*Pf* K1 CR	0,5	Brucein	0,005	0,002	0,00001	0,4	4
*Cryptolepis sanguinolenta* (Lindl.)	Apocynaceae	Pf K1 CR	5,41	Cryptolepine	0,054	0,04	0,002	7,7	5
*Diospyros sanza-minika* A. Chevalier	Ebenaceae	*Pf* K1 CR	0,8	4-O-(3′-methylgalloyl) norbergenin	0,6	1,2	0,01	3,1	6
*Erythrina fusca* Lour.	Fabaceae	*Pf* K1 CR	7,5	Citflavanone	5	0,1	0,01	0,4	7
*Erythrina fusca* Lour.	Fabaceae	*Pf* K1 CR	7,5	Lonchocarpol	1,6	0,2	0,02	1,9	7
*Erythrina fusca* Lour.	Fabaceae	*Pf* K1 CR	7,5	8-Prenyldaidzein	3,9	0,0006	0,00005	0,002	7
*Garcinia cowa* L.	Clusiaceae	*Pf* T9/94CS	5	7-O-Methylgarcinone	2,5	0,01	0,00030	0,02	8
*Garcinia cowa* L.	Clusiaceae	*Pf* T9/94CS	5	Cowanin	3	0,2	0,01	0,7	8
*Garcinia cowa* L.	Clusiaceae	*Pf* T9/94CS	5	Cowanol	1,6	0,5	0,03	3,1	8
*Garcinia cowa* L.	Clusiaceae	*Pf* T9/94CS	5	Vowaxanthone	1,5	0,4	0,02	2,6	8
*Garcinia cowa* L.	Clusiaceae	*Pf* T9/94CS	5	b-Mangostin	3	0,04	0,002	0,1	8
*Geissospermum sericeum* Miers	Apocynaceae	*Pf* K1 CR	1,78	Flavopereirine	2,84	0,04	0,0008	0,06	9
*Gomphostemma niveum* Hook. f.	Lamiaceae	*Pf* MR-C02 CS	9,7	Gomphostenin	38,2	0,5	0,05	0,3	10
*Gomphostemma niveum* Hook. f.	Lamiaceae	*Pf* MR-C02 CS	3,4	Gomphostenin-A	3,4	24	0,83	39	10
*Guiera senegalensis* J.F. Gmel.	Combretaceae	*Pf* W2 CR	4,45	Harman (b-carboline)	3,29	0,1	0,00445	0,3	11
*Holostylis reniformis* Duch*.*	Rubiaceae	*Pf* BHz26/86 CR	0,7	Lignan	0,12	0,4	0,003	4,6	12
*Holostylis reniformis* Duch.	Rubiaceae	*Pf* BHz CR	0,7	Lignan	0,12	4,5	0,03	42	12
*Nauclea orientalis* L.	Rubiaceae	*Pf* D6 CS	3	Oleanolic acid	4,6	0,07	0,002	0,08	13
*Phyllanthus niruri* L.	Euphorbiaceae	*Pf* CS	1,3	Terpenes	1,3	0,1	0,002	0,3	14
*Piptadenia pervillei Vatke* (Entada pervillei Vatke (R.Vig.)	Fabaceae	*Pf* MCF29	3,7	Catechin derivatives	0,4	0,03	0,001	0,6	15
*Piptadenia pervillei Vatke* (Entada pervillei Vatke (R.Vig.)	Fabaceae	Pf FcM29 CR	3,7	Catechin derivatives	0,3	0,1	0,004	2,4	15
*Pleiocarpa mutica* Benth.	Apocynaceae	*Pf* K1 CR	16,7	Pleiomutinine	3,2	0,05	0,008	0,5	16
*Polyalthia debilis* (Piere) Finet & ganep	Annonaceae	*Pf* K1 CR	1,35	Bis-dehydroaporphine	4,1	0,16	0,002	0,1	17
*Pothomorphe peltata* L.	Piperaceae	*Pf* K1 CR	3,7	4-Nerolidylcatechol	0,21	5,7	0,21	100	18
*Quassia amara* L.	Simaroubaceae	*Pf* W2 CR	8,9	Simalikalactone D	0,005	0,001	0,0001	3,5	19
*Rhaphidophora decursiva* Schott	Araceae	*Pf W2 CR*	6,8	Polysyphorin	0,37	0,00004	0,000003	0,001	20
*Rourea minor* (Gaertn.) Alston	Connaraceae	*Pf* W2 CR	2	Rourinoside (glycoside)	1,2	4	0,08	12,5	21
*Stephania pierrei* Diels	Menispermaceae	*Pf* W2 CR	3	Asimilobine	0,4	0,3	0,008	3,7	22
*Strychnos icaja* Baillon	Loganiaceae	*Pf W2 CR*	0,3	18-hydroxyisosungucine	0,09	0,03	0,0001	0,2	23
*Tapirira guianensis* Aubl.	Anacardiaceae	*Pf* F32 CR	18	Cyclic alkyl polyol derivatives	4,7	2,7	0,49	18,9	24
*Tephrosia elata* Deflers	Fabaceae	*Pf* D6 CS	8,4	Elatadihydrochalcone	2,8	0,2	0,02	1,1	25
*Tephrosia elata* Deflers	Fabaceae	*Pf* D6 CS	8,4	Obovatin	4,9	0,05	0,004	0,2	25
*Tephrosia elata* Deflers	Fabaceae	*Pf* D6 CS	8,4	Obovatin methyl ether	3,8	0,01	0,001	0,03	25
*Tephrosia elata* Deflers	Fabaceae	*Pf* D6 CS	8,4	Deguelin	6,3	0,01	0,001	0,02	25
*Teucrium ramosissimum* Desfontaines	Lamiaceae	*Pf* FCB1	2,7	Homalomenol	1,2	0,04	0,001	0,2	26
*Tithonia diversifolia* (Hemsl.) A. Gray	Asteraceae	*Pf* FCA20 Ghana CS	0,75	Tagitinin (toxic)	0,33	2,7	0,02	11,6	27
*Toddalia asiatica* (L.) Lam.	Rutaceae	*Pf* K39 CS	22	Coumarin	16,2	2,0	0,44	5,3	28
*Vernonia brasiliana* L.	Asteraceae	*Pf* BH2 CR	50	Lupeol	25	0,4	0,22	1,7	29
*Vernoniopsis caudata* (Drake) Humbert	Asteraceae	*Pf* FCB1 CR	1,6	Helenalin-[2-(1-hydroxyethyl)acrylate]	0,37	0,1	0,002	0,9	30
*Vernoniopsis caudata* (Drake) Humbert	Asteraceae	*Pf* FCB1 CR	1,6	Helenalin-[(2-hydroxyethyl-3-methyl)acrylate]	0,07	0,01	0,0002	0,5	30
*Vernoniopsis caudata* (Drake) Humbert	Asteraceae	*Pf* FCB1 CR	1,6	11R,13-dihydrohelenalin-[2-(1-hydroxyethyl)acrylate]	0,15	0,02	0,0003	0,4	30
*Viola verecunda* A. Gray	Violaceae	*Pf* FCB1 CR	25	Epioleanolic acid	0,18	0,03	0,01	7	31
*Zanthoxylum rhoifolium* Lam.	Rutaceae	*Pf* FCB1 CR	10	Nitidine	1,8	6,00	0,6	50	32
*Zhumeria majdae* Rech.f. & Wendelbo	Lamiaceae	*Pf* W2 CR	7,5	12,16-dideoxy aegyptinone B	1,4	0,6	0,05	6,2	33

The equation describing the relationship between concentration and IC_50_ is:

f=max-(max-min)/(1+x/EC_50_)^slope^ where “f” is the inhibitory effect. The EC_50_ is the IC_50_ of the isolated compound and “x” is the yield-dependent calculated concentration of the compound at the IC_50_ of the extract. For the simplest case, the values are set such that max=100 and max-min=100 and slope=1. The calculated partial effect of various compounds appears in the column captioned “% of active compound at extract IC_50_”.

Taking for example the case of the crude extract of *Alstonia macrophylla* and one of the most active compounds, macrocarpamine: one obtains a yield for macrocarpamine of 0.95 % and it is straightforward to calculate that it is present in the extract at 0.054 mg at the IC_50_ of the extract. Using the above equation one gets f=16.7. Thus, the active compound contributes 16.7/50 of the overall effect or 33.4 %. Another active compound, villalstonine, contributes 31.1 % to the activity of the crude extract. Given the fact that there are other active compounds in the extract, it is possible to suggest that the effects of macrocarpamine and villalstonine are not synergized in the crude extract and that their effects are additive. In such cases it can be concluded that very few active compounds account for the activity of the crude extract.

However, in the case of *Garcinia cowa* and 7-0-methylgarcinone, 0.0003 mg of the compound was present in the extract at the IC_50_ of the extract. The calculated f~0 and the compound contributes only 0.02 % to the anti-plasmodial activity of the crude extract. Since for all other purified compounds (cowanin, cowanol, cowaxanthone and b-mangostin) the contributions are ≤ 3.0 %, one is inclined to suggest that a strong synergism must occur between the components. Alternatively and quite unlikely, the extraction procedure destroys ***all*** the active compounds. In the extreme case of *Pothomorphe peltata* all the activity of the extract is accounted for by the activity of 4-nerolidylcatechol.

Inspection of the values that appear in the column captioned “% of active compound at extract IC_50_”, reveal that all cases can be subdivided in two groups. In one, the contribution of active compound to extract inhibition is ≥ ~20 %, while in the second the values center around ~1 % or significantly lower. Thus, in the second group considerable synergism between active compounds must exist in order to account for the activity of the extract, or the extraction procedure (quite unlikely) destroys the activity of all compounds.

Among the hundreds of articles describing the anti-plasmodial activity of plant extracts (1,031 articles were retrieved from PubMed for the last 10 years), only very few included the activity of the whole extract and of the pure compounds and their respective yields of extraction. Nevertheless it is striking that for 90% of the plants compiled in Additional file 1 the anti-malarial activity of the purified compounds cannot account quantitatively with that of the crude extract. If indeed this observation reflects the reality of anti-malarial properties of plant extracts, may be research should be focused on the “drug beside the drug”, looking for structures perhaps not exciting in the chemical point of view but that can revolutionize the treatment of malaria. Another natural consequence of this analysis is that evolution has provided not only bioactive metabolites that plants use to fight their foes, but has also mixed them in a very auspicious combination of compounds, which in some cases also work well in mammals. To achieve a similar combination even by systematic bioguided mixing is a very tedious, lengthy and expensive procedure. Why not learn from nature and optimize the use of plant extracts?

## Competing interests

The authors declare that they have no competing interests.
